# Urine metabolic profile changes of CCl_4_-liver fibrosis in rats and intervention effects of Yi Guan Jian Decoction using metabonomic approach

**DOI:** 10.1186/1472-6882-13-123

**Published:** 2013-06-03

**Authors:** Xiaojun Gou, Qing Tao, Qin Feng, Jinghua Peng, Yu Zhao, Jianye Dai, Wenyu Wang, Yongyu Zhang, Yiyang Hu, Ping Liu

**Affiliations:** 1 Institute of Liver Disease, Shuguang Hospital, Shanghai University of Traditional Chinese Medicine, Shanghai 201203, China; 2 Center for Traditional Chinese Medicine and Systems Biology of Shanghai University of Traditional Chinese Medicine, Shanghai 201203, China; 3 E-institute of Shanghai Municipal Education Commission, Shanghai 201203, China

**Keywords:** Yi Guan Jian Decoction, Carbon tetrachloride, Liver fibrosis, Urine metabolic profiles, GC/MS

## Abstract

**Background:**

Yi Guan Jian Decoction (YGJD), a famous Chinese prescription, has long been employed clinically to treat liver fibrosis. However, as of date, there is no report on the effects of YGJD from a metabonomic approach. In this study, a urine metabonomic method based on gas chromatography coupled with mass spectrometry (GC/MS) was employed to study the protective efficacy and metabolic profile changes caused by YGJD in carbon tetrachloride (CCl_4_)-induced liver fibrosis.

**Methods:**

Urine samples from Wistar rats of three randomly divided groups (control, model, and YGJD treated) were collected at various time-points, and the metabolic profile changes were analyzed by GC/MS with principal component analysis (PCA) and partial least squares-discriminate analysis (PLS-DA). Furthermore, histopathology and biochemical examination were also carried out to ensure the success of CCl_4_-induced liver fibrosis model.

**Results:**

Urine metabolic profile studies suggested distinct clustering of the three groups, and YGJD group was much closer to the control group by showing a tendency of recovering towards the control group. Fourteen significantly changed metabolites were found, and YGJD treatment could reverse the levels of these metabolites to normal levels or close to normal levels.

**Conclusions:**

The current study indicates that the YGJD has significant anti-fibrotic effects on CCl_4_-induced liver fibrosis in rats, which might be by regulating the dysfunction of energy metabolism, amino acid metabolism, tryptophan metabolism, cytochrome P450 metabolism, and gut microflora metabolism. The metabonomic approach can be recommended to study the pharmacological effect and mechanism of complex Chinese medicines.

## Background

Liver fibrosis is a characteristic of most types of chronic liver diseases. A number of factors such as viral infections (hepatitis B and C), alcoholism, autoimmune diseases and nonalcoholic fatty liver disease etc., may cause liver fibrosis [1], which corresponds to an increased production of extracellular matrix components(collagens in particular) and leads to liver dysfunction and cirrhosis [2,3]. With the involvement of progression and regression, liver fibrosis is a dynamic and bi-directional process [4]. However, cirrhosis, the end-stage consequence of fibrosis, is generally irreversible. Nevertheless, no curative treatment for liver fibrosis is available until today [5]. It is imperative to explore treatment options and discover more effective drugs against fibrosis and its severe consequences [6]. With a long history in the treatment of diseases, Traditional Chinese Medicine (TCM) are gaining popularity among patients with liver diseases and as effective putative antifibrotic agents [7,8].

Yi Guan Jian Decoction (YGJD), a classical TCM formula, is composed of *Radix glehniae, Radix ophiopogonis, Radix angelicae sinensis, Radix rehmannia, Fructus lycii* and *Fructus toosendan*. A recent study has indicated the hepatoprotective and antifibrotic effects of YGJD against dimethylnitrosamine-induced hepatic injury in rats. YGJD suppressed the elevation of serum glutamate oxaloacetate transaminase (GOT) and serum glutamic pyruvic transaminase (GPT) and significantly reduce the accumulation of collagen α1-I, tissue inhibitor of metalloproteinase −1(TIMP)-1 and alpha-smooth muscle actin (α-SMA) in liver tissues [9]. It significantly inhibited carbon tetrachloride (CCl_4_)-induced liver fibrosis and cirrhosis in rats, which might be associated with decrease in the liver content of hydroxyproline, α-SMA mRNA and Afamin mRNA expression [10,11]. Until now, a number of researches have been centered on the mechanism and efficacy of YGJD. But, little attention has been changed on the entire endogenous metabolites in YGJD processing organism. Moreover, the major active constituents of YGJD, ferulic acid and catalpol, significantly inhibited the progression of CCl_4_-induced hepatic fibrosis induced in rats [12]. These constituents may contribute to understand the protective efficacy and therapeutic mechanism of YGJD.

Metabonomics is a relatively new science and is an important branch of systems biology. It is defined as “*the quantitative measurement of the dynamic multiparametric metabolic response of living systems to pathophysiologic stimuli or genetic modification*” [13]. Metabonomics involves the analysis of endogenous metabolites of various biofluids and tissues, and harvests a latent relationship between the changed metabolic profiles and the physiological status of the biosystems [14]. This research strategy complies well with the integrity and systemic feature of TCM. Metabonomic technique has shown immense promise and has been applied to various domains [15], such as drug toxicity studies [16], drug efficacy studies [17], biomarker studies, and clinical diagnosis of diseases [18] etc. Some of the different analytical techniques currently in use include hydrogen-1 nuclear magnetic resonance, high-performance liquid chromatography/mass spectrometry, fourier transform infrared spectroscopy, and gas chromatography/mass spectrometry (GC/MS) [19]. Among them, GC/MS is a robust, unbiased method in identifying and quantifying metabolites with high sensitivity, simplicity, high reproducibility and NIST database accessibility [20,21]. It has gained the potential to become a powerful tool for metabonomic analysis of urine samples [22]. The present study investigated the urine metabolic profiles changes of CCl_4_-induced liver fibrosis in rats and studied the intervention effects of YGJD using a GC/MS based metabonomic technique. With the perturbed metabolites, attempts were made to explore the possible therapeutic mechanism of YGJD.

## Methods

### Reagents

Ethyl chloroformate, pyridine, anhydrous ethanol, sodium hydroxide, chloroform, CCl_4_, olive oil, isopropanol, anhydrous sodium acetate, methanol and anhydrous sodium fulfate were of analytical grade and were obtained from China National Pharmaceutical Group Corporation (Shanghai, China). L-2-chlorophenylalanine (Shanghai IntechemTech. Co. Ltd., China) was used as an internal quality standard. Hydroxyproline was from Japan ナ カ Te ィ Te Su Ku Ltd., (Tokyo, Japan), and perchloric acid was from Shanghai Jinglu Chemical Co. Ltd., (Shanghai, China). Commercial kits used for determining AST, ALT, Alb, GGT and TBil were obtained from Nanjing Jiancheng Institute of Biotechnology (Nanjing, China).

### YGJD Preparation

YGJD was prepared using the method proposed by Yongping Mu *et al.*[12]. The following six dried raw herbs were purchased from Shanghai Huayu Chinese Herbs Co. Ltd., (Shanghai, China): 1.0 kg of *Radix glehniae*, 1.0 kg of *Radix ophiopogonis*, 1.0 kg of *Radix angelicae sinensis*, 1.8 kg of *Radix rehmannia*, 1.2 kg of *Fructus lycii* and 0.45 kg of *Fructus toosendan*. The mixture was decocted twice with boiling water (1.5 h each time). The decoction was then filtered, mixed and concentrated under reduced pressure to fluid extract, which was dried under vacuum to afford 4.27 kg of dry powder and stored in cold preservation until use.

### Animals

Male Wistar rats of weight 140 g to 150 g were obtained from Shanghai Experimental Animal Center of Chinese Academy of Sciences. Animal foods were commercially obtained from Shanghai Laboratory Animal Center. (Shanghai, China). All rats were housed individually under set temperature (20 ± 5°C) and humidity (50 ± 10%) with a 12/12 h-light/dark cycle. The animals were provided with common food and water ad libitum. The experimental procedures were approved by the Ethics Committee of the Institute of Shanghai University of TCM.

### CCl_4_-induced live fibrosis

After an initial acclimation period of 2 weeks in cages, CCl_4_ (1 mL/kg 50% CCl_4_, diluted in olive oil, twice each week) was injected intraperitoneally (IP)for 9 weeks to induce rat live fibrosis as described by Nakamura T and colleagues [23].

### Treatment groups

After treatment with CCl_4_ for 6 weeks, the model rats were randomly divided into 2 groups: model group (n = 10), YGJD group (n = 11). YGJD group was oral administrated at dosage 2.682 g/kg body weight of YGJD, diluted in water, intragastric administration (IG), once a day for 3 weeks, and concomitantly received CCl_4_ treatment. A control group rats (n = 6) and model group were orally administrated with same volume of saline solution.

### Samples collection

Urine samples at 12 h were collected during week 0 before CCl_4_ injection and week 1, 6, 8 and 9 after CCl_4_ injection. All collected urine samples were immediately stored at -80°C before GC/MS analysis. After the final collection time point, all animals were anesthetized with sodium pentobarbital (2 mL/kg, IP) and sacrificed by cervical dislocation. Blood samples were obtained from the abdominal aorta and centrifuged at 3000 rpm for 10 min to obtain serum. Livers were isolated and stored at −80°C for pathological observation.

### Analysis of liver function

Serum alanine aminotransferase (ALT), aspartate aminotransferase (AST), total bilirubin (TBil), albumin(Alb) and γ-glutamyl traspeptidase(GGT) were determined to assess liver function using commercially available kits (Nanjing Jiancheng Institute of Biotechnology, China) according to manufacturer’s instructions.

### Measurement of hepatic hydroxyproline contents

Hepatic hydroxyproline contents were measured by the method of Jamall and colleagues [24], and the results were expressed as μg/mg wet tissue.

### Histology

A portion of each liver was fixed in 10% neutral formalin, dehydrated and embedded in paraffin. The samples were stained with hematoxylin and eosin (HE) and sirius red staining for histological examination per standard procedure. Fibrosis was graded by Li C *et al.*[25] as follows: grade 0-no fibrosis, normal liver; grade I-fibrosis present; grade II-mild fibrosis; grade III- moderate fibrosis; and grade IV- severe fibrosis.

### Urine sample preparation and GC/MS analysis

The urine sample preparation and GC/MS analysis were performed as described by Qiu Y *et al.*[26], which were shown in the Additional file 1.

### Data analysis

All raw data were converted to document format, and then processed by the XCMS (http://metlin.scripps.edu/download/) with default settings to carry out baseline correction, peak discrimination and alignment. The result was exported into Microsoft Excel 2007, where each peak was normalized to the total sum of peaks. The resulted three-dimensional data involving the peak number, sample name, and normalized peak area were fed to SIMCA-P 11.5 software package(Umetrics, Umea, Sweden) for principal component analysis (PCA) and partial least squares discriminant analysis (PLS-DA) after undertaking a unit variance procedure*.* The concentrations of the significantly changed metabolites were represented as their relative areas (divided by the area of internal standard).

### Statistics

Quantitative data was presented as means +/− SD. Statistical analysis was analyzed by one-way analysis of variance with Student–Newman–Keuls test using the SPSS17.0 software (SPSS, Chicago, USA. Histological grade from the liver were evaluated using Ridit analysis. P < 0.05 was considered statistically significant.

## Results

### Liver function tests in serum

**Figure 1:**
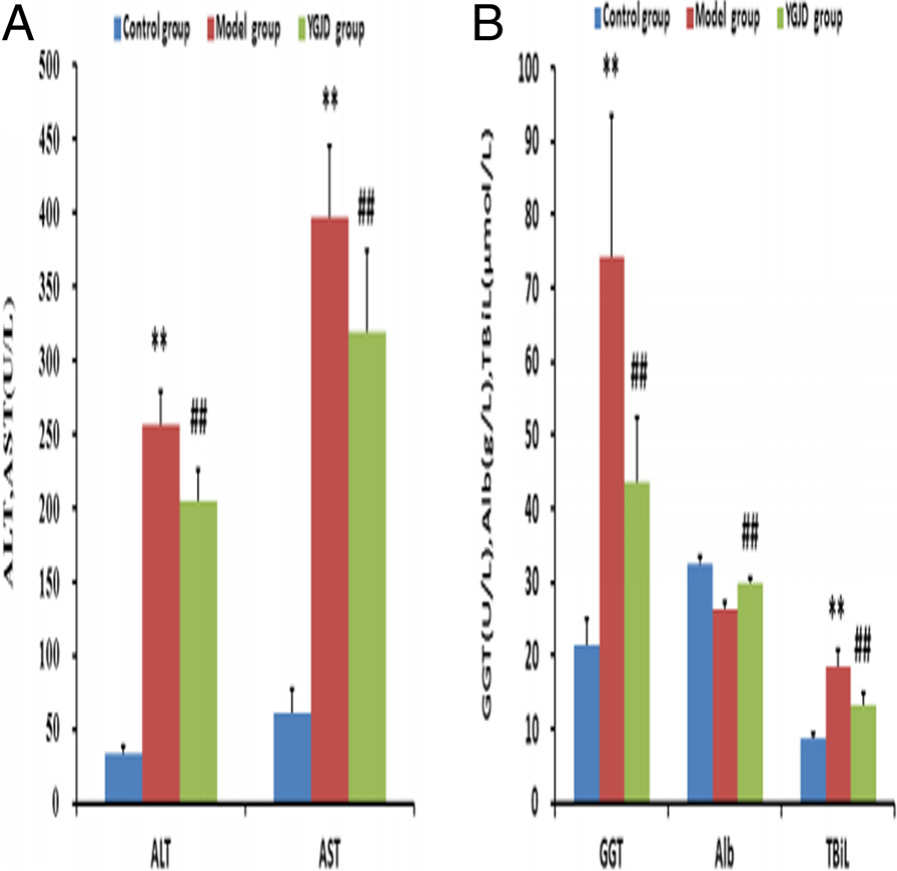
**Effect of YGJD on serum levels of AST, ALT (A) and GGT, Alb, TBiL (B) in CCl_4_-induced liver fibrosis in rats.** Values are expressed as mean ± SD. ** compared with the control group, P < 0.01; ^##^ compared with model group, P < 0.01.

As shown in Figure 1, the levels of AST, ALT, GGT and TBil in serum were significantly increased in model group compared with control group (P < 0.01), but they were significantly decreased in the YGJD group compared with model group (P < 0.01). Serum Alb content in model group was significantly lower than that in the control group (P < 0.01). YGJD could elevate Alb content and there was significant difference compared with the control group (P < 0.01).

### Hepatic hydroxyproline content

**Figure 2:**
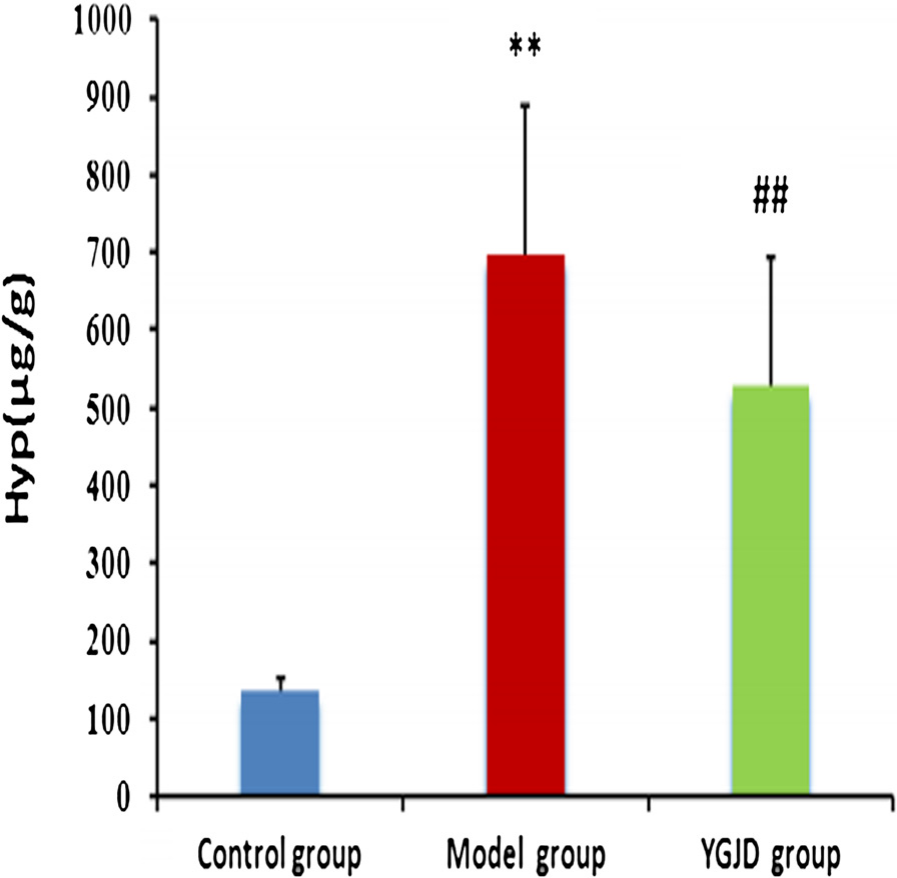
**Effect of YGJD on the hepatic hydroxyproline content in liver fibrosis rats.** Values are the mean ± SD. ** P < 0.01 compared with the control group; ^##^ P < 0.01 compared with the model group.

In model group, hepatic hydroxyproline content was approximately increased 5 times over the control group (P < 0.01). However, the hydroxyproline concentration was significantly decreased in YGJD group after YGJD administeration (P < 0.01) (Figure 2).

### Histological changes

**Figure 3:**
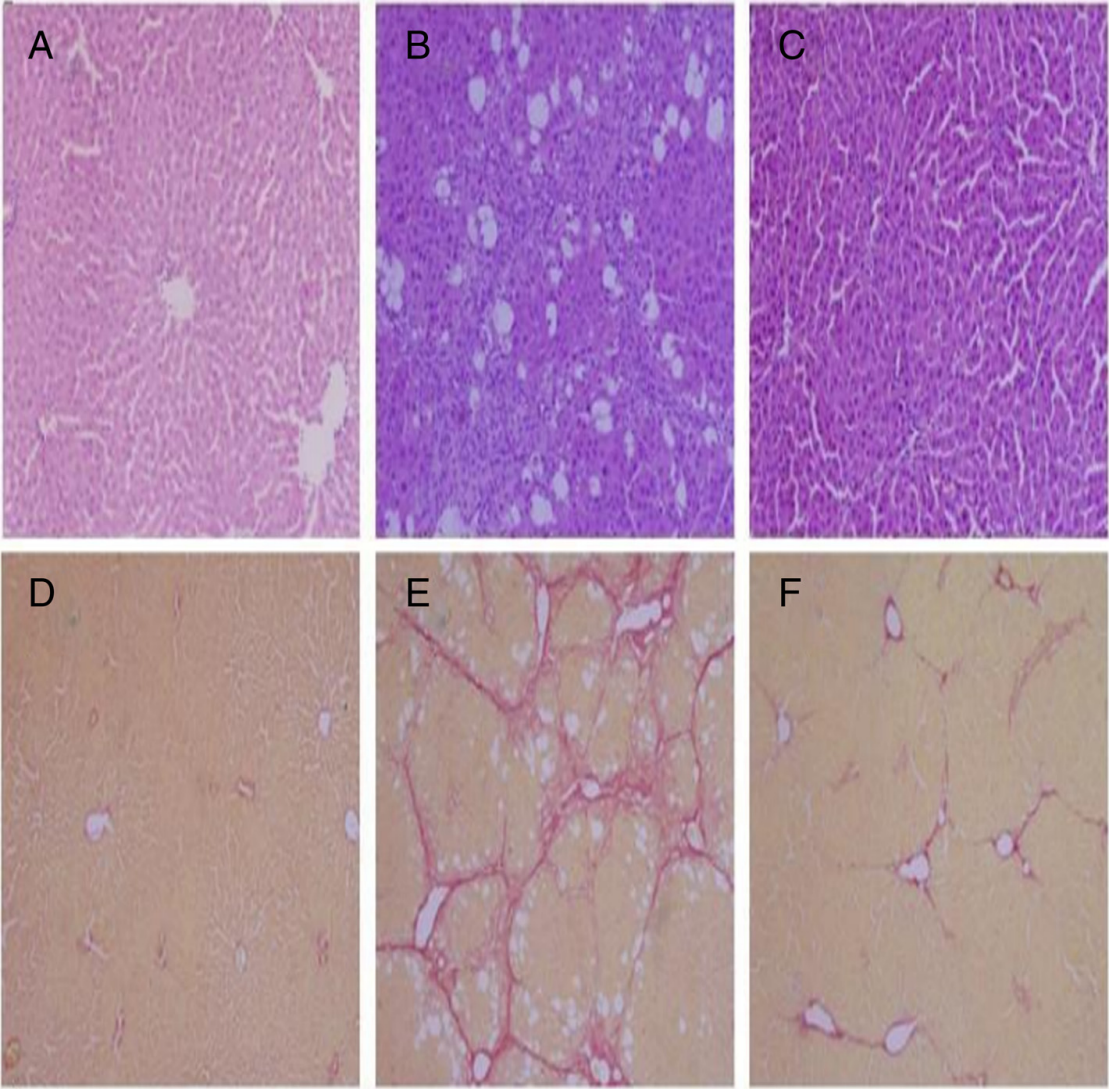
**Histological changes of liver tissues as shown by HE staining (×200) and Sirius Red staining (×200).** (**A**)-(**C**) HE staining; (**A**) Control group, (**B**) Model group, (**C**) YGJD group; (**D**)-(**F**) Sirius Red staining; (**D**) Control group, (**E**) Model group, (**F**) YGJD group.

As shown in Figure 3, HE staining and sirius red staining of liver sections were observed for histological changes. Liver tissue samples from the control group showed an intact liver tissue structure with little collagen deposition (Figure 3A and D). The liver tissue samples from the model group exhibited more fatty degeneration, more steatosis, cell necrosis, and infiltration of inflammatory cells and there were more collagen deposition compared with the control group (Figure 3B and E). In YGJD group, however, liver fibrosis symptoms were all apparently ameliorated and collagen deposition was also markedly reduced compared with the model group, (Figure 3C and F). The model group rats had a high degree of fibrosis compared with the control group (P < 0.05), and treatment with YGJD significantly decreased histological grade in YGJD group compared with the model group (P < 0.05) (Table 1).

**Table 1:** Effect of YGJD on the pathologic grading of CCl_4_-induced liver fibrosis in rats

**Group**	**n**	**Pathologic grading of liver fibrosis**					**P value**
		**0**	**I**	**II**	**III**	**IV**	
Control group	6	6	0	0	0	0	-
Model group	10	0	0	0	6	4	a
YGJD group	11	0	0	7	4	0	b

Statistical analysis of the data for comparisons of the pathological grading was performed by Ridit analysis; Figures represent number of rat per grade; a: P < 0.05, compared with control group; b: P < 0.05, compared with model group.

### Metabonomics

1) GC/MS spectra of the three groups

**Figure 4:**
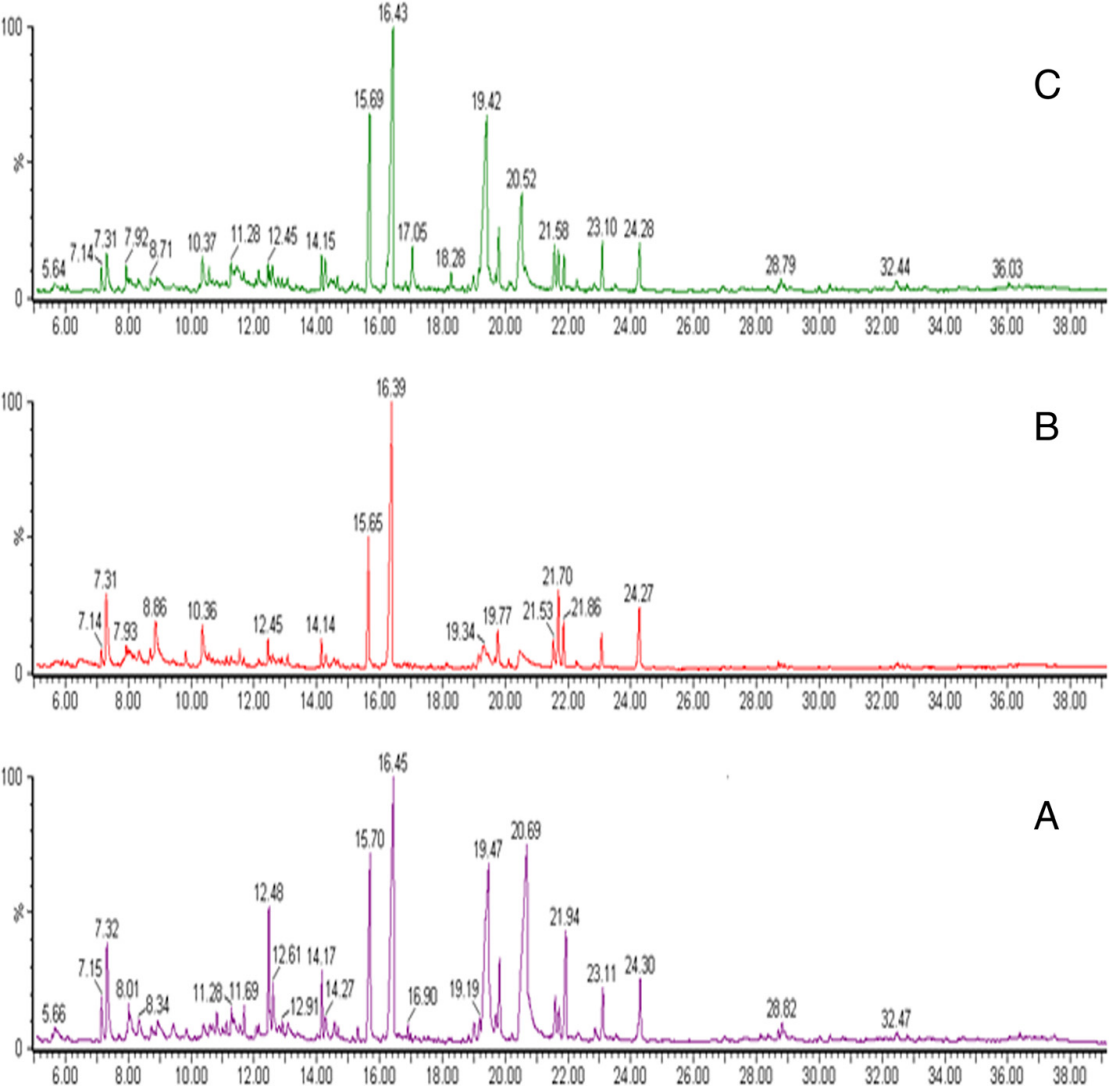
**Typical GC/MS TIC chromatograms of rat urine samples obtained from the three groups. A**: control group, **B**: model group, **C**: YGJD group.

The typical GC/MS total ion current (TIC) chromatograms of rat urine on 9th week from the control, model and YGJD groups are shown in Figure 4. There are obvious changes in both the control and model groups, while, the spectra are similar between control group and YGJD group. Based on NIST database and reference standards, the most peaks were identified as endogenous metabolites, which including the following: amino acids, organic acids and fatty acids. These metabolites were mainly involved in energy metabolism, lipid metabolism and amino acid metabolism. In order to illustrate the differences of the metabolic profiles, GC/MS spectra were further pre-treated and a pattern recognition analysis was carried out.

2) Analysis of metabolic profiles

In order to understand the general trends, differences and outliers among three groups by GC/MS spectra, the 9th week sample was selected owing to the greatest degree of pathological changes in rats and a PCA was carried out. The result showed unsatisfactory separation in the scores plot among three groups (figure not shown).

**Figure 5:**
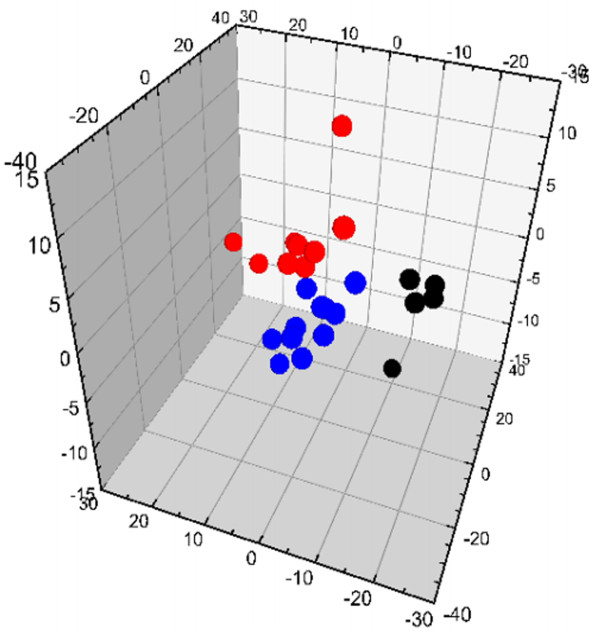
Score plot of 3D-PLS-DA model obtained from control group (●), model group (red bullet) and YGJD group (blue bullet).

**Figure 6:**
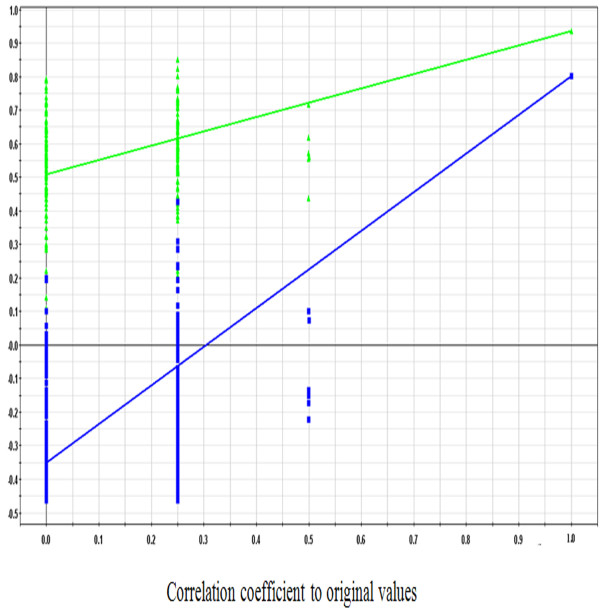
**Two hundred permutations were performed, and the resulting R^2^ and Q^2 ^ values were plotted.** (Green triangle): R^2^; (blue square): Q^2^. The green line represents the regression line for R^2^ and the blue line for Q^2^.

To obtain a higher level of group separation and enhance recognition of variables responsible for classification, a supervised PLS-DA was applied. The obtained 3D-PLS-DA score plot (Figure 5) showed better separation than PCA among three groups. From the 3D-PLS-DA score plot, a separation of the model group and control group was clearly achieved. The YGJD group was mainly located between the model group and the control group, and it exhibited a tendency to recover to control group. A 7-fold cross-validation was used to estimate the robustness and the predictive ability of our model [27], and the parameters for the classification from the software were R^2^Y = 0.90 and Q^2^Y = 0.48, which were stable and good to fitness and prediction, respectively. A permutation test was proceeded in order to further validate the model [28]. The R^2^ and Q^2^ intercept values were 0.51 and −0.35 after 200 permutations. The negative values of Q^2^ intercept indicate the robustness of the models, and thus show a low risk of over fitting and reliable (Figure 6). These results demonstrate that YGJD treatment could restore the disturbed metabolic profiles of rats.

3) Changed metabolites analysis of urine samples

Based on the3D-PLS-DA, a loading plot was constructed, which showed the contribution of variables to difference among three groups. It also showed the important variables which were situated far from the origin but the loading plot is complex because of many variables (figure not shown).

To refine this analysis, the variable importance projection (VIP) was obtained [28]. The VIP values exceeding 1.0 were first selected as changed metabolites. In step 2, the remaining variables were then assessed by *t* test, P > 0.05, variables were discarded between the model and control groups. Additionally, significantly changed metabolites detected were identified by using the reference compounds available and the commercial compound database of NIST. Meanwhile, the metabolites were interpreted for related pathway with the available biochemical databases, such as KEGG (http://www.genome.jp/kegg/), HMDB (http://www.hmdb.ca/). On the basis of the above analysis, 14 variables representing individual metabolites as significantly changed metabolites were listed out. These metabolites are shown in Table 2.

In particular, compared with the control group, the model group displayed increased levels of metabolites, including butanedioic acid, indole-3-carboxylic acid, citrate, hippuric acid, glutamate, and hexadecanoic acid, and decreased levels of glycine, leucine, phenol, proline, oleic acid, octadecenoic acid, lysine, and tryptophan. Furthermore, it was shown that the concentration alterations of the metabolites in YGJD group were all properly regulated when compared with the model group, and there were non-significant except for octadecenoic acid and lysine when compared with the control group. These results confirmed that the disturbed urine metabolite profiles owing to CCl_4_ exposure were regulated by YGJD. The results of liver function tests, histological changes*,* and these change in urine metabolic pattern showed that liver fibrosis was being prevented and alleviated after taking YGJD.

4) Time-dependent change of metabolic profile in YGJD group

**Figure 7:**
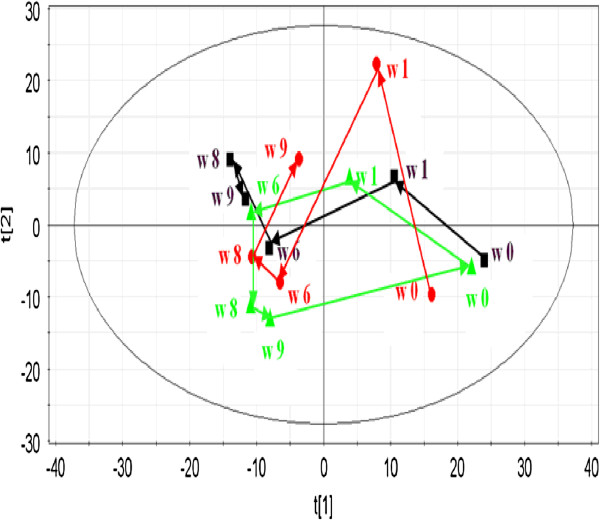
**Score plot from mean scores value of PCA derived from the GC/MS profiles of urine samples obtained from rats with control group (■), model group (red bullet) and YGJD group (green triangle) at weeks 0, 6, 8 and 9.** The plot depicts a time- related trajectory of metabolite patterns at different time points.

The time-related trajectory of metabolic patterns were obtained from the mean scores value of PC1 and PC2 at week 0 before CCl_4_ injection, week 1, 6, 8, and 9 after CCl_4_ injection (Figure 7). In the scores plot of PCA, no apparent changes of metabolic profile were observed in the control group. In the model group, the metabolic pattern at different time points showed distinct differences, and a tendency of deviating from time point of week 0 pre-dose, to week 9 post-dose was noted, which manifested the CCl_4_-induced metabolic alterations. In the YGJD group, the metabolic pattern of week 1 post-dose obviously deviated from that of week 0 pre-dose. The metabolic patterns on week 8 and week 9 showed the reversion tendency towards the week 0 pre-dose state with the treatment of YGJD. This outcome suggested that YGJD has the potential to correct those deviations induced by CCl_4_ exposure.

**Table 2:** Identification results of metabolites in this study

**Metabolites**	**Model group vs control group**	**YGJD group vs model group**	**YGJD group vs control group**	**Pathways**
Indole-3-carboxylic acid	↑*	↓	NS	Tryptophan metabolism
Butanedioic acid	↑*	↓#	NS	Energy metabolism
Glycine	↓*	↑	NS	P450 metabolism
Leucine	↓*	↑	NS	Amino acid metabolism
Phenol	↓*	↑	NS	Flora metabolism
Proline	↓**	↑#	NS	Amino acid metabolism
Citrate	↑*	↓##	NS	Energy metabolism
Hippuric acid	↑*	↓#	NS	P450 metabolism
Glutamate	↑*	↓	NS	Amino acid metabolism
Hexadecanoic acid	↑*	↓#	NS	Fatty acid metabolism
Oleic acid	↓*	↑	NS	Fatty acid metabolism
Octadecenoic acid	↓*	↑	△	Fatty acid metabolism
Lysine	↓**	↑	△	Amino acid metabolism
Tryptophan	↓*	↑#	NS	Tryptophan metabolism

“↓” indicates relative decrease, “↑” indicates relative increase, *P < 0.05, ** P < 0.01 compared with control group; ## P < 0.01, # P < 0.05 compared with model group; △P < 0.05 compared with control group; NS indicates Non-significant. Results were expressed as $\left(\bar{x}+/-\mathrm{SE}\right)$, units were mg/mL urine sample, which are provided in the Additional file 1.

## Discussion

Liver fibrosis occurs as a consequence of dynamic wound-healing response to acute or chronic hepatocellular damage(s), and it pose a high threat with significant morbidity and mortality [29]. Currently, no acceptable therapeutic strategies exist. There is a huge need and great significance to search for effective ways to inhibit liver fibrosis and prevent the development of cirrhosis [30]. The present study demonstrated that YGJD, a standardized extract of a TCM formula, had therapeutic effects on CCl_4_-induced liver fibrosis in rats.

An animal model of CCl_4_-induced liver fibrosis was established, and *in vivo* anti-fibrotic effects of YGJD were investigated. The histological results showed that the normal structure of lobules was destroyed, and pseudolobules were formed. Moreover, the increased hydroxyproline content in liver, the key characteristic component of collagen [31], also confirmed the hepatic fibrogenesis in rats. There was a significant increase in the levels of ALT, AST, GGT, TBil as well as decrease in serum Alb content on exposure to CCl_4_, indicating considerable hepatocellular injury. YGJD effectively reduced the elevated levels of hydroxyproline content, serum ALT, AST, GGT and TBil, and enhance the reduced serum Alb levels which were lower in CCl_4_-treated rats. The histopathological analysis suggested that YGJD obviously alleviated the degree of CCl_4_-induced liver fibrosis. Our earlier study showed that effect of YGJD on liver fibrosis was associated with its ability to improve the activity of matrix metalloproteinase (MMP)-9 and contents of MMP-13, TIMP-2 and hepatocyte growth factor alpha (HGFalpha) and decrease the activity of MMP-2 and contents of α-SMA, TIMP-1, caspase-12 and hepatocyte apoptotic index [12]. In addition, it also manifested that YGJD blocked the increase of transforming growth factor beta (TGF-β), and up-regulation of procollagen alphaI (data not shown). YGJD contains key bioactive compounds that include ferulic acid and catalpol. The present study showed that sodium ferulate (a sodium salt of ferulic acid) markedly inhibited HSC activation and collagen production, increased MMP-1 expression, and decreased TIMP-1expression [32]. Another study has reviewed that catalpol prevents d-galactose induced mitochondrial dysfunction in mice involving inhibiting of nitric oxide synthase activity, production of reactive oxygen species, and increase respiratory complex activities and MMP level [33]. The above data indicated that the potential of YGJD as an effective drug against hepatic fibrosis.

Apart from the biochemical and histological effects of YGJD, the GC/MS coupled with pattern recognition analysis were studied, and changes in urine metabolic profile were explored. The results of GC/MS-based metabonomic analysis of urine samples indicate that YGJD administration has a clear impact on the CCl_4_-induced metabolite disorder and can redress the perturbation of metabolites. These significantly changed metabolites may be explain the action mechanism of YGJD.

**Figure 8:**
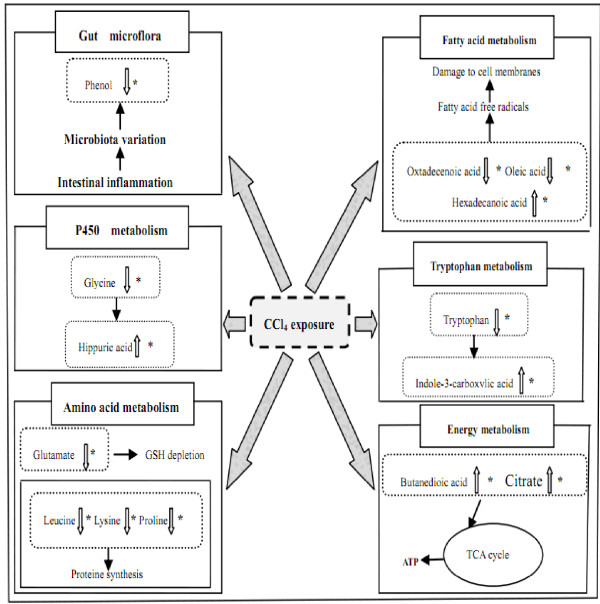
**The perturbed metabolic pathways in response to CCl_4_ exposure and YGJD treatment.** The levels of changed metabolites in model group compared to control group were labeled with (↓) down-regulated or (↑) up-regulated; *metabolites in abnormal could be regulated by YGJD.

Butanedioic acid and citrate are the intermediates of tricarboxylic acid (TCA) cycle and provide an easy energy supply for the body. In this study, butanedioic acid and citrate were obviously increased in model group compared with those in the control group, suggesting the dysfunction of energy metabolism. Oxidative stress has been shown to be a major molecular mechanism involved in CCl_4_ toxicity [34] and is connected with chronic liver diseases of many causes [35]. In the presence of oxidative stress, mitochondrial TCA cycle is slowed down in that cellular regulation reduce the generation of free radicals [36]. We infer that the increase of butanedioic acid and citrate is due to the dysfunction of TCA. It has been reported that catalpol, one of the active compounds of YGJD, is a natural component of *Rehmannia glutionsa*, has protective effects on energy metabolism disturbance [37]. Our previous study showed that YGJD improved hepatic glucose metabolism [38]. In this work, YGJD intervention evidently decreased the levels of butanedioic acid and citrate, and it recovered the tendency towards the normal levels, indicating that YGJD may protect against CCl_4_-induced fibrosis by regulation perturbations of energy metabolism (Figure 8).

As displayed in Table 2, the free fatty acids such as hexadecanoic acid, oleic acid, and octadecenoic acid, were significantly changed in model group compared with the control group. It has been reported that some free fatty acids had strong cytotoxicity, which may impair the cell membrane, mitochondria, and lysosomal membranes, inducing intracellular micro-organ damage, and significantly improve the toxicity of cytokines, leading to the liver degeneration, inflammatory cell infiltration and fibrosis. This indicates that the formation of CCl_4_–induced liver fibrosis is closely related to the changes of free fatty acids [39,40]. Our results demonstrated YGJD treatment could restore the altered levels of these three metabolites in model group rats towards those in control group rats. Ferulic acid, another active compound of YGJD, is isolated from *Angelica sinensis*, has potential antioxidant capacity and terminate free radical chain reactions,and effectively scaveng deleterious radicals [41]. Moreover, ferulic acid can prevent cell damage caused by O2-, and in particular by -OH and NO, in living systems [42]. From the above literature and results, it is well understood that fatty acids are important for the recovery of damaged livers in rats, and YGJD alleviates the consequences of liver fibrosis that may be associated with abnormalities in free fatty acid metabolisms (Figure 8).

In the model group, the glycine level was significantly decreased, and hippuric acid was significantly increased when compared with the control group (Table 2). Glycine is essential for hippuric acid production in the liver along with benzoic acid under cytochrome P450 catalysis [43]. Therefore, the alterations of hippuric acid and glycine concentrations in the model group indicate the changes in the activity of cytochrome P450 to some extent, and cytochrome P450 activity and liver fibrosis formation has a high degree of correlation [4]. In our research, YGJD regulated the metabolite level of glycine and hippuric acid. The results of histology and metabonomics demonstrated that the anti-fibrotic effect of YGJD might be associated with its activity of cytochrome P450 (Figure 8).

In the model group, leucine, lysine, and proline were significantly decreased, and, glutamate was significantly increased than in the control group, which suggested the abnormality of amino acid metabolism. It is reported that liver diseases are often associated with inflammation and oxidative stress, and these conditions facilitate the formation of advanced glycation end products, which are known to impair protein function and promote inflammation [44]. Amino acids are substrates for protein synthesis [45]. When the liver fibrosis models were made in this study, CCl_4_ intoxication could be attenuated amino acid uptake and proteins synthesis [46]. Leucine, is one of the branched-chain amino acids (BCAA) [47]. BCAA-enriched nutrients were found to reduce oxidative stress and stimulate antioxidant DNA repair in a rat model of CCl_4_-induced liver injury [48]. In addition, glutamate is one of three amino acids of the GSH biosynthesis, and GSH is a major antioxidant, which quenches the endogenous oxidant species and attacks exogenous oxidative stress and has been seen as a major molecular mechanism in CCl_4_ toxicity [49]. Previous studies reported that ferulic acid protects from CCl_4_-induced acute liver injury through reduction of oxidative damage and inflammatory signaling pathways [50]. YGJD intervention of CCl_4_-treated rats showed a tendency of bringing the level of leucine, lysine, proline, and glutamate to normal level or close to normal level. Based on these findings, it is likely that the antifibrotic effect of YGJD might be concerned with its antioxidative activity through modulating the perturbed amino acid metabolism pathway (Figure 8).

Tryptophan is an essential amino acid which cannot be synthesized by the body, and it must be drawn from the food. It is the precursor of serotonin, an important neurotransmitter, and it plays a big role in the process of protein synthesis and is involved in some pathological processes [45,51]. Previous research showed tryptophan administration promotes the reversion of CCl_4_- induced, pre-established chronic liver injury_,_ and suggests that tryptophan exerts this effect by enhancing several liver dysfunction parameters associated with chronic liver injury and also by stimulating hepatic protein synthesis [52]. In our study, the level of tryptophan in the urine is significantly lower in model group than control group. The tryptophan pathway could possibly be changed during the formation of liver fibrosis. Furthermore, indole-3-carboxylic acid level in the urine of model rats was markedly elevated compared with that of control rats. Up-regulation of indole-3-carboxylic acid might be the result of the tryptophan abnormal metabolism [53]. In YGJD group, the concentration of tryptophan and indole-3-carboxylic acid returned close to normal, indicating the therapeutic effects of YGJD may rely on the regulation of the dysfunction of tryptophan metabolism (Figure 8).

Phenol is considered as one the metabolites of gut microflora and derives from the bacterial aromatic amino acids and polyphenols fermentation that were from polyphenols in food and food protein decomposition [54]. Gut microflora play a big role in nutrition and disease prevention, and there is an intensive connection between liver and gut. Abnormal bile due to liver damage leads to the dysfunction of intestinal barrier,and thus, may cause the changes in intestinal flora or composition, or even bacterial translocation and its metabolite changes [55]. A recent publication reported that the gut microbiota alterations were associated with the development of an inflammatory environment, fibrosis progression and bacterial translocation in CCl_4_-treated mice [56]. Changes in these endogenous metabolites might probably be due to gut microflora alterations caused by CCl_4_ exposure. In clinical studies, *Escherichia coli Nissle* were administered to patients with liver cirrhosis, which confirmed that probiotics might reduce the level of endotoxin and improve liver functions. The restoration of normal bacterial flora in the gut resulted in lower absorption of toxic metabolites and endotoxins in treated patients [57]. In our work, in the model group, phenol was significantly decreased compared with the control group. YGJD group has higher levels of phenol compared with the model group. In conclusion, CCl_4_ could affect gut microflora and change endogenous metabolites. From the results, it is suggested that YGJD may exert its anti-fibrotic efficacy by regulating the gut flora (Figure 8).

## Conclusions

This is the first study to evaluate the urine metabolic profile changes of CCl_4_-induced liver fibrosis in rats and examine the intervention effects of YGJD through a metabonomic based GC/MS method. Multivariate statistical analysis showed that the separation of model and control groups was clearly achieved. YGJD group was mainly located between these two groups and exhibited a tendency of recovering to control group. Fourteen significantly changed metabolites related to antifibrotics by YGJD were identified, and they could be reversed by YGJD treatment. This indicated that the therapeutic effect of YGJD on liver fibrosis may involve in regulating the dysfunction of energy metabolism, amino acid metabolism, tryptophan metabolism, cytochrome P450 metabolism, and gut microflora metabolism. This work confirmed that a metabonomic method can be used to study the efficacy and mechanism of complex TCM in a dynamic and non-invasive way. Further investigations using different animal models and hepatotoxic agents are recommended in the future using metabonomic approach to get more data on the reliability of YGJD in chronic liver diseases.

## Abbreviations

CCl4: Carbon tetrachloride; YGJD: Yi Guan Jian Decoction; GC/MS: Gas chromatography coupled to mass spectrometry; GOT: Glutamate oxaloacetate transaminase; GPT: Glutamic pyruvic transaminase; TIMP-1: Tissue inhibitors of metalloproteinases-1; α-SMA: α-smooth muscle actin; TCM: Traditional Chinese medicine; ALT: Alanine aminotransferas; AST: Aspartate aminotransferase; Alb: Albumin; TBil: Total bilirubin; GGT: γ-glutamyltranspetidase; H&E: Hematoxylin-eosin; PCA: Principal component analysis; PLS-DA: Partial least squares-discriminate analysis; VIP: Variable influence on projection; TIC: Total ion current; TCA: Tricarboxylic acid; HGF alpha: Hepatocyte growth factor alpha; TGF-β: Transforming growth factor beta; BCAA: Branched-chain amino acids.

## Competing interests

The authors declare that they have no competing interests.

## Authors’ contributions

YYH, YYZ and PL conceived the study, reviewed the results, and critically reviewed the manuscript. JGP and QF carried out the experiments. JYD, YZ and WYW performed the study and analyzed the data. XJG and QT participated in the study design, analyzed the data, and contributed to drafting the manuscript. All authors read and approved the final manuscript.

## Pre-publication history

The pre-publication history for this paper can be accessed here:

http://www.biomedcentral.com/1472-6882/13/123/prepub

## Supplementary Material

Additional file 1 Urine sample preparation and identification results of metabolites. Click here for file
