# Supplementary material for: Urine metabolic profile changes of CCl4-liver fibrosis in rats and intervention effects of Yi Guan Jian Decoction using metabonomic approach
Source: BMC Complement Altern Med. 2013 Jun 3;13:123. doi: 10.1186/1472-6882-13-123 (PMC3680307; doi:10.1186/1472-6882-13-123)
Supplement: Additional file 1 — Urine sample preparation and identification results of metabolites. [file 1472-6882-13-123-S1.pdf]

Urine Sample preparation

About 600 μL of urine sample was added to a screw-top glass tube. The following were then added to the urine sample:100 μL of L-2-chlorophenylalanine (0.10 mg/mL, internal standard for batch quality control), 400 μL of anhydrous ethanol, and 100 μL of pyridine. Ethyl chloroformate (ECF, 50 μL) was added for first derivatization at 20.0 ±0.1 °C with sonication at 40 kHz for 60 seconds. Subsequently, the content was extracted using 300 μL of chloroform with the aqueous layer PH carefully adjusted to 9–10 using 100 μL of sodium hydroxide (7mol/L). The derivatization procedure was repeated with the addition of 50 μL ECF into the aforementioned products. After the two successive derivatization steps, the contents were vortexed for 30 seconds and centrifuged for 3 min at 3000 rpm. The aqueous layer was aspirated off; while the remaining chloroform layer containing the derivatives were isolated and dried with anhydrous sodium sulfate. The isolated derivatives were subjected to GC/MS analysis.

GC/MS analysis

Each 1 μL aliquot of analytes was injected into a DB-5MS capillary column coated with 5% diphenyl-95% dimethylpolysiloxane (30 m × 250 μm ID, 0.25 μm film thickness, Agilent J&W Scientific, Folsom, CA) and 6890N Agilent gas chromatography (GC, Agilent Technologies, Palo Alto, CA) connected to an Agilent 5975C mass spectrometer (Agilent Technologies, Palo Alto, CA) in the split less mode. The injection and the interface temperature were set at 260 ℃, and the ion source was adjusted to 200 ℃. Helium was used as the

carrier gas (flow rate: 1 mL/min). Details of the oven temperature program were as follows: Initial GC oven temperature was 80 ℃, 2 min after injection. The GC oven temperature was raised to 140 ℃ with 10 ℃/min, then to 240 ℃ at a rate of 4 ℃/min, to 280 ℃ with 10 ℃ /min again, and finally held at 280 ℃ for 3 min. The electron energy was 70 eV, and analytes were detected in full scan mode (m/z 30-550).

Table 2

Identification results of metabolites in this study

Metabolites	Model group	Control group	YGJD group	Pathways
Indole-3-carboxylic acid	0.346 ±0.053	0.168 ±0.037	0.300±0.058	Tryptophan metabolism
Butanedioic acid	1.119 ±0.145	0.442 ±0.073	0.622±0.092	Energy metabolism
Glycine	0.145 ±0.051	0.456 ±0.129	0.219 ±0.077	Amino acid metabolism
Leucine	0.044 ±0.018	0.117 ±0.027	0.058 ±0.015	P450 metabolism
Phenol	0.137 ±0.011	0.203 ±0.026	0.155 ±0.014	Flora metabolism
Proline	1.090 ±0.19	2.233 ±0.207	2.280 ±0.430	Amino acid metabolism
Citrate	1.424±0.191	0.744 ±0.212	0.766 ±0.095	energy metabolism
Hippuric acid	0.251±0.065	0.051 ±0.009	0.067 ±0.019	P450 metabolism
Glutamate	0.476±0.141	0.271±0.146	0.237±0.056	Amino acid metabolism
Hexadecanoic acid	0.270±0.063	0.066±0.036	0.064±0.032	Fatty acid metabolism
Oleic acid	0.175±0.015	0.247±0.034	0.319±0.094	Fatty acid metabolism
Octadecenoic acid	0.051 ±0.022	0.159 ±0.040	0.074 ±0.019	Fatty acid metabolism
Lysine	0.067 ±0.021	0.236±0.053	0.112 ±0.028	Amino acid metabolism
Tryptophan	0.097±0.037	0.314±0.081	0.324 ±0.071	Tryptophan metabolism

Results units were expressed（$\bar{x}$ +/-SE）， units were mg/mL urine sample.
